# Diet and Exercise Interventions among Overweight and Obese Lactating Women: Randomized Trial of Effects on Cardiovascular Risk Factors

**DOI:** 10.1371/journal.pone.0088250

**Published:** 2014-02-07

**Authors:** Hilde K. Brekke, Fredrik Bertz, Kathleen M. Rasmussen, Ingvar Bosaeus, Lars Ellegård, Anna Winkvist

**Affiliations:** 1 Department of Internal Medicine and Clinical Nutrition, Sahlgrenska Academy at the University of Gothenburg, Gothenburg, Sweden; 2 Division of Nutritional Sciences, Cornell University, Ithaca, New York, United State of America; 3 Department of Clinical Nutrition, Sahlgrenska University Hospital, Gothenburg, Sweden; University of British Columbia, Canada

## Abstract

**Objective:**

To examine the effects of Diet (D) and Exercise (E) interventions on cardiovascular fitness, waist circumference, blood lipids, glucose metabolism, inflammation markers, insulin-like growth factor 1 (IGF-1) and blood pressure in overweight and obese lactating women.

**Methods:**

At 10–14 wk postpartum, 68 Swedish women with a self-reported pre-pregnancy BMI of 25–35 kg/m^2^ were randomized to a 12-wk behavior modification treatment with D, E, both or control using a 2×2 factorial design. The goal of D treatment was to reduce body weight by 0.5 kg/wk, accomplished by decreasing energy intake by 500 kcal/d and monitoring weight loss through self-weighing. The goal of E treatment was to perform 4 45-min walks per wk at 60–70% of max heart-rate using a heart-rate monitor. Effects were measured 12 wk and 1 y after randomization. General Linear Modeling was used to study main and interaction effects adjusted for baseline values of dependent variable.

**Results:**

There was a significant main effect of the D treatment, decreasing waist circumference (*P* = 0.001), total cholesterol (*P* = 0.007), LDL-cholesterol (*P* = 0.003) and fasting insulin (*P* = 0.042), at the end of the 12-wk treatment. The decreased waist circumference (*P*<0.001) and insulin (*P* = 0.024) was sustained and HDL-cholesterol increased (*P* = 0.005) at the 1-y follow-up. No effects from the E treatment or any interaction effects were observed.

**Conclusions:**

Dietary behavior modification that produced sustained weight loss among overweight and obese lactating women also improved risk factors for cardiovascular disease and type 2 diabetes. This intervention may not only reduce weight-related risks with future pregnancies but also long-term risk for metabolic disease.

**Trial registration:**

ClinicalTrials.gov NCT01343238

## Introduction

Cardiovascular disease (CVD) is the main cause of death in women in Sweden [1] and most western societies, including the US [2]. Important risk factors for CVD in women include dyslipidemia, hypertension, diabetes, obesity and low fitness [3]. Childbearing is also associated with risk of CVD [4]–[6] and the metabolic syndrome [7] in epidemiological studies. Weight retention from childbearing is one important contributor to this risk [5] although changes in body composition [8], lipid metabolism [9] and lifestyle [5] have been suggested as additional pathways. Overweight and obese women who have born children are a group at risk for future development of obesity-related metabolic disease. Interventions that promote weight loss can potentially mitigate this risk.

The possibility of implementing lifestyle changes during the postpartum period has gained interest because many women are motivated to lose the extra weight gained during pregnancy [10] and the responsibility of being a parent may stimulate interest in a healthy living [11]. In short-term studies performed in the US, physical exercise and dietary restrictions alone or combined did not have negative effects on the quantity or quality of breast milk or on infant growth [12]–[14].

The effect of lifestyle intervention postpartum on plasma lipids and other metabolic variables has only been investigated in few studies. Lovelady et al. [15] concluded that there was no effect of exercise alone on blood lipids or resting metabolic rate in normal-weight women during lactation although VO_2_ max increased by 25%. However, weight loss was small and did not differ between the exercise group and controls. Stendell-Hollis et al. [16] found a significant decrease in TNFα when comparing Mediterranean-style and MyPyramid diets for weight loss postpartum although no significant between-group difference was found. Thus, the effects of diet intervention alone or in combination with exercise on cardiometabolic risk factors in overweight and obese women undergoing significant weight reduction are not known. These lifestyle interventions would be the obvious therapeutic options to counteract cardiometabolic risk factors in overweight and obese women if supported by controlled clinical trials.

In a randomized clinical factorial trial in Sweden, we have shown that 12 wk of dietary behavior modification produced a safe and clinically significant weight loss of 9.0% in overweight and obese lactating women that was sustained at 10.0% at the 1-year follow-up [17]. There was no significant main effect of exercise behavior modification on body weight. Weight loss among women not receiving the dietary treatment was 1.8% and 2.0% after 12 wk and 1 y, respectively. Metabolic changes from this sustained weight loss could be important for women’s long term health considering the increased risk of cardiovascular disease and the metabolic syndrome in childbearing women [4]–[7]. Here we examine the 12-wk and 1-y effects of diet and exercise as well as their possible interactions on measures of the cardiovascular risk factors cardiovascular fitness, waist circumference, blood lipids, glucose metabolism, inflammation markers, insulin-like growth factor 1 (IGF-1) and blood pressure among women who participated in this randomized trial.

## Materials and Methods

### Participants and Setting

The LEVA study (Swedish: Livsstil för Effektiv Viktminskning under Amning, in English: Lifestyle for effective weight loss during lactation), a randomized controlled 2×2 factorial trial, examined the effects of diet, exercise or both on several outcomes. **Trial registration:** http//www.clinicaltrials.gov NCT01343238. The protocol for this trial and supporting CONSORT checklist are available as supporting information; see Checklist S1 and Protocol S1.

The outcomes of this intervention on body weight and body composition after the 12-wk treatment period and at the 1-y follow-up have been reported [17].

Women who were overweight or obese class I (BMI 25–34.9 kg/m^2^) before pregnancy (self-reported data) were recruited during pregnancy or up to 8 wk postpartum from 15 antenatal care clinics in Gothenburg, Sweden, during 2007–2010. Midwives informed eligible women about the study during visits to antenatal care or eligible women responded to advertising with posters at the antenatal-care clinics. To be included in the study women had to be non-smoking, have the intention to breastfeed for at least 6 mo, and provide <20% of the baby’s estimated energy intake from non-breast milk sources. The babies had to be singleton, full-term with a birth weight >2500 g. Both mother and infant should be free of serious illness. Women medicated for hypothyroidism or mild allergies were eligible. The LEVA study was approved by the Regional Ethical Review Board, Gothenburg, Sweden. Written informed consent was provided by all participants.

### Study Design and Randomization

Participants were randomized to 1 of 4 groups; control (no treatment) (C), dietary behavior modification (D), physical exercise behavior modification (E) or dietary and physical exercise behavior modification (DE) at 10 to 14 wk postpartum. Randomization was stratified by BMI ≥28.0 or <28.0 kg/m^2^ using a blocked randomization with a block size of 4 women within each stratum. Group allocation was revealed after baseline measurements were complete. In those randomized to treatment, the 12-wk intervention was initiated shortly thereafter.

### Examinations

Baseline examinations were performed twice between 8–12 wk postpartum and took about 2 wk to complete due to urine collection for doubly labeled water analysis. At the first examination, cardiovascular fitness and blood pressure were measured. At the second examination the remaining measurements were performed [2.3±4.4 (mean ± SD) d between examinations]. Women came to the laboratory after a 7–10 h overnight fast (no food or drinks, except water, after midnight). They were instructed to drive or use public transportation and avoid strenuous activities before arriving at the laboratory. All measurements were repeated after the 12-wk treatment (5–6 mo postpartum) and all measurements except total energy expenditure were repeated again at the 1-y follow-up (15 mo postpartum).

### Measurements

#### Waist circumference

Waist circumference was measured to the nearest 0.5 cm mid-way between the lower margin of the last palpable rib and the top of the iliac crest [18].

#### Blood chemistry

Blood samples were sent for immediate analysis of plasma glucose, serum insulin and insulin-like growth factor 1 (IGF-1). Serum was frozen (−70°C) to allow for analysis of each participant’s blood lipids (total cholesterol, triglycerides, HDL-cholesterol, LDL-cholesterol) and high sensitive C-reactive protein (hs-CRP) from before and after intervention in the same batch to reduce measurement errors. These analyses were performed at the Central Laboratory at the Sahlgrenska University Hospital. Two markers of inflammation (IL-6 and TNF-α) were measured in serum with the Human Proinflammatory 9-plex Ultrasensitive Kit in a SECTOR 2400 Imager (Meso Scale Discovery, Gaithersburg, MD) at the Wallenberg Laboratory for Cardiovascular and Metabolic Research, University of Gothenburg, Sweden.

#### Cardiopulmonary exercise test

A bicycle ergometer test (EBIKE Comfort, GE Medical System, Milwaukee, WI) was performed in upright position until exhaustion and reaching a minimum respiratory exchange ratio of 1∶1. The workload started at 40 W and increased with 15 W per min. Breath-by-breath gas analysis by Ergospirometry was applied (Jaeger Oxycon Pro, Viasys Healthcare GmbH, Hoechenberg, Germany) and VO_2_ max was measured as the highest oxygen uptake during the test.

#### Blood pressure

Blood pressure was measured after 5 min of rest in the supine position prior to the cardiopulmonary exercise test using the auscultatory method on the right arm.

### Treatments

Detailed description of the diet and exercise treatment protocols have previously been published [17].

#### Control group (C)

Women who were randomized to the control group were instructed to live as usual regarding diet and physical activity.

#### Dietary behavior modification intervention (D)

The goal in the dietary intervention was to achieve a weight reduction of 6 kg during the 12-wk intervention period at the rate of 0.5 kg/wk. The diet group received a total of 2.5-h individual dietary behavioral intervention with a registered dietitian. The dietary intervention was based on the 4-d food diary and designed to reduce energy intake with 500 kcal/d with a nutrient composition according to current nutrition recommendations [19]. Participants were instructed to follow a stepwise plan to introduce dietary change, one change at a time to facilitate weekly and final weight-loss goals. Body weight was self-monitored using a digital scale (Arko, EKS, Gislaved, Sweden) provided by the study. For reinforcement participants were asked to report the latest measured body weight in a cell-phone text message every 2 wk and received feedback on their performance.

#### Physical exercise behavior modification intervention (E)

The goal of group E was to perform a 45-min walk at 60–70% of maximal heart rate (“pulse zone walk”) 4 times weekly during the 12-wk intervention period. Group E received a total of 2.5-h individual behavioral intervention from a physical therapist. Participants were provided with a heart rate monitor (Polar FS2C, Polar Electro Oy, Kempele, Finland) and instructed how to perform the walks within the recommended pulse range (as determined during cardiovascular fitness test). For reinforcement participants were asked to report the number of pulse zone walks performed during the previous wk in a cell-phone text message every 2 wk and received feedback on their performance.

#### Dietary and physical exercise behavior modification intervention (DE)

The combined group received both dietary and physical exercise interventions as described above, for a total of 5 h.

### Statistical Analysis

A sample size of 68 was chosen based on expected results on weight change [17]. Power analyses for secondary outcomes were not possible to perform as this study was exploratory and no suitable data were available. Baseline characteristics among the 4 groups were compared using one-way ANOVA, Chi-square test and Kruskal-Wallis test. General Linear Modeling, adjusted for baseline values of the dependent variable, was used to analyze main effects of diet, main effects of exercise and possible interaction effects (ranks were used for non-normally distributed variables). All analyses were performed on an intention-to-treat basis. For IL-6 and TNFα, values>+3 SD were removed before analyses. A *P* value of <0.05 was considered statistically significant. SPSS (version 21.0, IBM, Somers, NY) was used for all statistical analyses.

## Results

**Figure 1:**
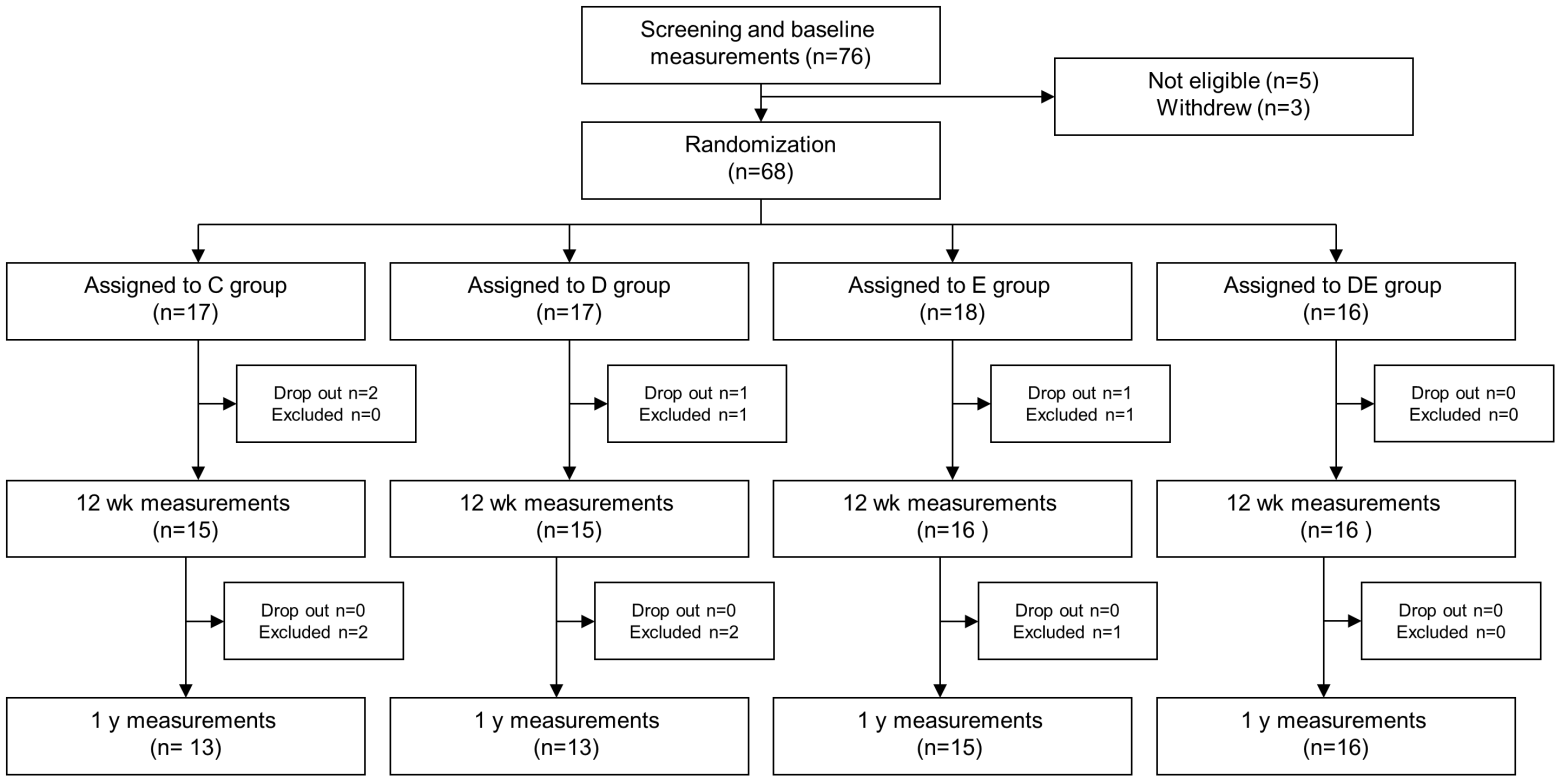
Subject-flow diagram of the LEVA trial. C denotes control, D dietary behavior modification, E exercise behavior modification, and DE combined dietary plus exercise behavior modification.

Results are presented for the 62 women (91%) who completed both the baseline and 12-wk measurements as well as the 57 women (84%) who also completed the 1-y follow-up; see **Figure 1** for subject flow diagram. Two women were excluded during the intervention; 1 because of new pregnancy and 1 because of new medication, while 4 dropped out for other reasons. Between intervention and 1-y follow-up, 5 women were excluded because of new pregnancies and none dropped out. During the intervention, participants in D treatment (D+DE groups) reported weighing themselves at least twice weekly. Participants in E treatment (E+DE groups) completed 83% of planned walks.

The participating women were on average 33±4 (mean ± SD) y of age, weighed 86±10 kg with a BMI of 30±3 kg/m^2^. The majority (53%) were primiparous; 42% and 5% had 2 and 3 children, respectively. There were no statistically significant differences in maternal characteristics among study groups at baseline [17] except for slightly lower fasting plasma glucose in the dietary treatment groups (**Table 1**). No differences were observed in breastfeeding behavior or indicators of infant growth between groups before or after intervention [17].

**Table 1:** Measurements at baseline in overweight and obese, lactating women participating in the LEVA study by intervention group^1^.

	Control (n = 15)	Diet (n = 15)	Exercise (n = 16)	Diet+Exercise (n = 16)	*P*-value
Cardiovscular risk outcomes					
Waist circumference, cm	98 (10)	94 (7)	98 (10)	95 (7)	0.444
VO_2_ max, L/min	2.19 (0.34)^2^	2.14 (0.31)	2.26 (0.30)	2.20 (0.42)	0.839
Diastolic blood pressure, mm Hg	74 (7)^2^	76 (8)	77 (9)^3^	79 (6)	0.459
Systolic blood pressure, mm Hg	113 (12)^2^	119 (11)	118 (10)	117 (9)	0.465
Fasting Plasma glucose, mmol/L	4.8 (0.39)	4.3 (0.53)	4.6 (0.30)	4.4 (0.28)	0.004
Fasting Serum insulin mU/L	7.0 (3.7)	5.4 (3.2)	5.8 (2.2)	5.5 (2.4)	0.410
Total-C, mmol/L	5.1 (0.76)	4.9 (0.77)	5.0 (0.98)	5.1 (0.95)	0.927
LDL-C, mmol/L	3.1 (0.75)	2.8 (0.70)	3.1 (0.87)	3.1 (0.82)	0.659
HDL-C, mmol/L	1.6 (0.28)	1.7 (0.21)	1.5 (0.25)	1.5 (0.31)	0.191
LDL-C/HDL-C	2.1 (0.72)	1.7 (0.42)	2.2 (0.80)	2.1 (0.77)	0.188
Triglycerides, mmol/L	0.84 (0.34)	0.68 (0.20)	0.88 (0.33)	0.77 (0.37)	0.327
IGF-1, µg/L	149 (31)	141 (51)	155 (24)	149 (37)	0.756
Inflammation markers^4^					
hs-CRP, mg/L	2.3 (1.5, 5.0)	3.0 (1.3, 6.1)	2.1 (1.2, 2.8)	1.7 (0.84, 4.6)	0.430
IL-6, pg/mL	1.02 (0.64, 1.33)	0.91 (0.58, 1.10)	0.86 (0.69, 1.19)	0.81 (0.62, 1.29)	0.867
TNF- α, pg/mL	8.75 (6.27, 12.1)	9.44 (7.17, 10.6)	9.96 (8.00 12.0)	8.31 (6.71, 10.8)	0.489

1 For normally distributed variables, values are means (SD) and one-way ANOVA was used for comparisons among groups at baseline. For non-normally distributed variables (inflammation markers), values are medians (inter quartile range) and Kruskal-Wallis test was used for comparisons among groups at baseline.

2 No valid data retrieved for 1 woman in the Control group (n = 14).

3 No data retrieved for 1 woman in the Exercise group (n = 15).

4 One woman in the Control group with CRP of 22.0 mg/L and signs of breast engorgement and hence local inflammation was excluded from analysis (n = 14). One woman in the Diet group and one woman in the Exercise group were excluded (IL-6 and TNF-α values>+3 SD) (n = 14 and n = 15, respectively).

BP: blood pressure, HDL-C: High density lipoprotein cholesterol, hs-CRP: high sensitive C-reactive protein, IGF-1: Insulin-like growth factor 1, IL-6: Interleukin 6, LDL-C: Low density lipoprotein cholesterol, TNF-α: Tumor necrosis factor-alpha, Total-C: Total cholesterol.

### Main and Interaction Effects of Treatments on Cardiovascular Risk Outcomes

There was a main effect of the D treatment reducing waist circumference at 12 wk (*P* = 0.001) and 1 y (*P*<0.001) (**Table 2**).

**Table 2:** Effect of diet, exercise and interaction on clinical indicators among overweight and obese, lactating women after 12 wk intervention and 1-y follow-up^1^.

	Control	Diet	Exercise	Diet+Exercise	*P* main effect Diet	*P* main effect Exer-cise	*P* inter-action
	Δ	Δ	Δ	Δ			
	n = 15^2^	n = 15^2^	n = 16^2^	n = 16			
Waist circumference, 12 wk	−1.3 (5.6)	−8.7 (5.6)	−4.3 (7.0)	−8.8 (4.1)	0.001	0.923	0.428
Waist circumference, 1 y	−3.9 (5.6)	−10.7 (5.8)	−6.0 (9.9)	−9.5 (6.2)	<0.001	0.320	0.225
Cardiovascular fitness^3^							
VO_2_ max, L/min, 12 wk	0.17 (0.19)	−0.00 (0.26)	0.18 (0.18)	0.08 (0.23)	0.014	0.364	0.372
VO_2_ max, L/min, 1 y	0.23 (0.13)	0.17 (0.24)	0.21 (0.25)	0.19 (0.19)	0.238	0.913	0.658
Blood lipids							
Total-C, mmol/L, 12 wk	−0.35 (0.68)	−0.78 (0.47)	−0.44 (0.47)	−0.62 (0.72)	0.007	0.701	0.145
Total-C, mmol/L, 1 y	−0.75 (0.60)	−0.68 (0.34)	−0.71(0.59)	−0.56 (0.72)	0.571	0.286	0.337
LDL-C, mmol/L, 12 wk	−0.24 (0.66)	−0.56 (0.46)	−0.28 (0.40)	−0.47 (0.53)	0.003	0.431	0.230
LDL-C, mmol/L, 1 y	−0.46 (0.68)	−0.44 (0.32)	−0.47 (0.47)	−0.39 (0.60)	0.870	0.212	0.254
HDL-C, mmol/L, 12 wk	−0.05 (0.18)	−0.13 (0.15)	−0.02 (0.19)	−0.05 (0.23)	0.388	0.549	0.736
HDL-C, mmol/L, 1 y	−0.26 (0.30)	−0.11 (0.13)	−0.19 (0.19)	−0.06 (0.24)	0.005	0.909	0.664
LDL-C/HDL-C, 12 wk	−0.13 (0.50)	−0.20 (0.31)	−0.19 (0.47)	−0.23 (0.34)	0.092	0.579	0.387
LDL-C/HDL-C, 1 y	0.02 (0.68)	−0.17 (0.24)	−0.08 (0.32)	−0.19 (0.42)	0.029	0.406	0.271
Triglycerides, mmol/L, 12 wk	0.02 (0.27)	−0.09 (0.19)	−0.13 (0.40)	−0.11 (0.36)	0.097	0.495	0.232
Triglycerides, mmol/L, 1 y	0.15 (0.52)	0.03 (0.21)	0.06 (0.25)	−0.01 (0.37)	0.072	0.912	0.725
Glucose homeostasis							
Fasting glucose, mmol/L, 12 wk	−0.08 (0.50)	−0.01 (0.55)	−0.07 (0.35)	−0.02 (0.29)	0.079	0.552	0.328
Fasting glucose, mmol/L, 1 y	0.22 (0.55)	0.36 (0.72)	0.11 (0.33)	0.26 (0.32)	0.486	0.176	0.384
Fasting insulin, mU/L, 12 wk	0.42 (2.9)	−0.56 (3.1)	0.58 (1.5)	−0.29 (3.1)	0.042	0.956	0.599
Fasting insulin, mU/L, 1 y	2.45 (3.8)	0.46 (2.80)	1.94 (1.13)	1.05 (2.42)^4^	0.024	0.957	0.370
Inflammation markers^5^							
hs-CRP, mg/L, 12 wk	−0.77 (−1.9, 0.65)	−0.66 (−3.5, 0.01)	−0.50 (−1.52, −0.07)	−0.42 (−1.17, −0.11)	0.644	0.593	0.799
hs-CRP, mg/L, 1 y	−1.2 (−2.8, −0.38)	−1.5 (−4.0, −0.33)	−0.71 (−1.49, −0.14)	−0.40 (−0.80, −0.10)^4^	0.743	0.338	0.381
IL-6, pg/mL, 12 wk	0.13 (−0.15, 0.46)	−0.12 (−0.29, 0.28)	−0.14 (−0.41, 0.18)	−0.11 (−0.42, 0.07)	0.086	0.079	0.202
IL-6, pg/mL, 1 y	−0.04 (−0.62, 0.19)	−0.22 (−0.37, 0.25)	−0.15 (−0.28, 0.12)	−0.17 (−0.73, 0.19)	0.313	0.720	0.634
TNF- α, pg/mL, 12 wk	−0.00 (−0.88, 0.74)	−0.32 (−1.03, 0.49)	−0.53 (−1.33, 1.37)	−0.23 (−2.29, 0.96)	0.534	0.901	0.998
TNF-α, pg/mL, 1 y	0.18 (−0.15, 1.49)	−1.28 (−2.27, 0.94)	−1.0 (−3.07, 0.00)	−1.80 (−2.35, 0.06)	0.246	0.178	0.630
Blood pressure^3^^,^ 6							
Systolic BP, mm Hg, 12 wk	0.8 (9.3)	−7.5 (8.1)^7^	−2.6 (13)	−4.1 (14)	0.209	0.773	0.517
Systolic BP, mm Hg, 1 y	2.4 (16)	−10 (8.1)	−3.0 (11)	−4.8 (8.7)	0.080	0.669	0.254
Diastolic BP, mm Hg, 12 wk	−5.6 (7.8)	−6.1 (11)	−7.5 (11)	−6.2 (8.6)	0.730	0.632	0.852
Diastolic BP, mm Hg, 1 y	−2.1 (10)	−2.9 (9.9)	−4.2 (9.6)	−6.1 (8.5)	0.730	0.618	0.976
Other							
IGF-1, 12 wk	17.5 (37.9)	26.5 (42.6)	30.0 (27.1)	22.1 (30.5)	0.931	0.672	0.348
IGF-1, 1 y	44.9 (44.2)	100.3 (81.6)	58.0 (48.2)	55.6 (48.1)^4^	0.067	0.230	0.059

1 Mean (SD) for changes in normally distributed variables and median (inter quartile range) for changes in non-normally distributed variables (inflammation markers). General Linear Modeling for main and interaction effects, adjusted for baseline values of dependent variable (ranks were used for non-normally distributed variables).

2 At the 1-y follow-up the number of women was reduced in groups to: Control: n = 13, Diet: n = 13 and Exercise: n = 15.

3 No valid data retrieved for 2 women in the Control group at 12 wk and 1 y (n = 13 and n = 11, respectively) and 1 woman in the Exercise group at 1 y (n = 14) as well as 1 woman in the Diet and Exercise group at 1 y (n = 15).

4 No value obtained for 1 woman in the Diet and Exercise group (n = 15).

5 One woman in the control group with CRP of 22.0 mg/L and signs of breast engorgement and hence local inflammation at baseline was excluded from analysis (n = 14 and n = 12 at 12 wk and 1 y, respectively). Exclusions (IL-6 and TNFα values>+3 SD): One woman in the Diet group at 12 wk and 1 y (n = 14 and n = 12, respectively), one woman in the Exercise group at 12 wk (n = 15), one woman in the Diet and Exercise group at 1 year (IL-6 only) (n = 15).

6 No Diastolic BP retrieved for 2 women in the Diet group at 12 wk (n = 11), 2 and 3 women in the Exercise group at 12 wk (n = 13) and 1 y (n = 12) respectively and 2 women in the Diet and Exercise group respectively (n = 14 and n = 14 at 12 wk and 1 y, respectively).

7 No Systolic BP retrieved for 1 woman in the Diet group (n = 14).

BP: blood pressure, HDL-C: High density lipoprotein cholesterol, hs-CRP: high sensitive C-reactive protein, IGF-1: Insulin-like growth factor 1, IL-6: Interleukin 6, LDL-C: Low density lipoprotein cholesterol, TNF-α: Tumor necrosis factor-alpha, Total-C: Total cholesterol.

At 12 wk, there was a negative main effect of the D treatment on VO_2_ max (*P* = 0.014) as the groups not receiving D treatment increased their VO2 max. At 1 y there was no longer any main effect of D treatment on VO_2_ max.

**Figure 2:**
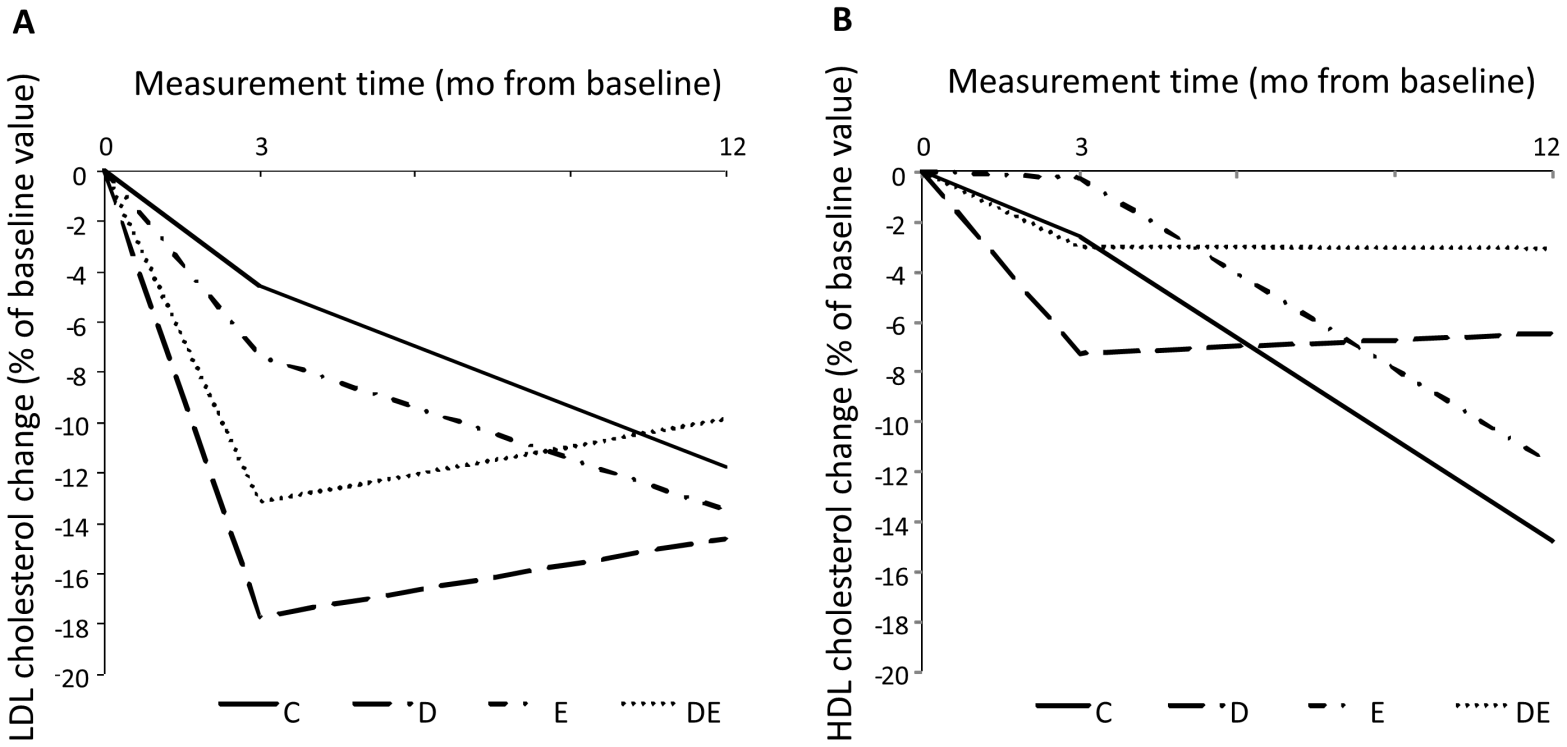
Effect of interventions on change in LDL-cholesterol (panel A) and HDL-cholesterol (panel B). No statistical comparisons were made between groups as shown in the figures. C denotes control, D dietary behavior modification, E exercise behavior modification, and DE combined dietary plus exercise behavior modification.

As expected, cholesterol values generally decreased during the 12-wk intervention period when most women were still breastfeeding. However, there was still a main effect of the D treatment reducing total cholesterol (*P* = 0.007) and LDL-cholesterol (*P* = 0.003) (Table 2 and **Figure 2A**). At 1 y, these effects were no longer evident; in contrast HDL cholesterol was maintained higher (*P = *0.005) (**Figure 2B**) and the ratio of LDL to HDL cholesterol remained lower (*P* = 0.029) as a main effect of D treatment.

Fasting insulin decreased as a main effect of the D treatment at 12 wk (*P* = 0.042) and 1 y (*P = *0.024). Fasting plasma glucose, resting systolic and diastolic blood pressure, inflammation markers and IGF-1 were not significantly affected by the D treatment.

No main effects of the E treatment were observed in VO_2_ max, blood lipids, fasting plasma glucose or serum insulin, systolic or diastolic blood pressure, inflammation markers or IGF-1 (all *P* -values >0.05, Table 2).

No interaction effects of the treatments were observed in any of these cardiovascular risk outcomes.

## Discussion

This study shows that dietary treatment that caused significant and sustained weight reduction in lactating and overweight obese women also caused sustained improvements in cardiovascular risk factors, such as waist circumference and concentrations of blood lipids and fasting insulin. Interventions resulting in exercise of higher intensity, if tolerated at this time postpartum, are likely needed to achieve beneficial effects on cardiovascular fitness among relatively active women like those in LEVA.

Blood lipids are elevated during pregnancy and decrease postpartum [20] depending on the intensity of breastfeeding [21]. Our participants were likely close to their pre-pregnant concentrations of these lipids when our intervention began at 12 wk postpartum [20]. A reduction in total- and LDL-cholesterol of 4–6% occurred in the LEVA control group during the 12-wk intervention. Therefore, the observed reductions of 11–18% in total- and LDL-cholesterol values in the D groups are greater than could be expected from partial lactation alone. Interestingly, the difference in LDL-cholesterol reduction between the D group and controls is about twice that predicted from the difference in weight loss between the groups [22].

HDL-cholesterol is known to decrease during active weight loss and increase when subjects stabilize at a lower weight [22]. This may explain the observed long-term increase in HDL-cholesterol (*P*<0.01) of about 15% (0.2 mmol/l or 4.3 mg/dL) in the D treatment groups relative to the non-diet groups (figure 2B). The positive effect of our intervention on HDL-cholesterol is of special interest because childbearing is associated with a decrease in HDL-cholesterol values of about 3–4 mg/dL, with the effect persisting 10 y later [9]. A difference in HDL-cholesterol of 1 mg/dL corresponds to a difference in cardiovascular disease risk of about 3% in women [23]. This suggests that the diet intervention in LEVA has the potential to reduce the risk of CVD in women by 12%. It is also noteworthy that although breastfeeding is known to have an “HDL-maintaining” effect compared to non-breastfeeding, [24] our groups not receiving dietary treatment (C+E) but still breastfeeding had a steep downward trajectory for their HDL-cholesterol. Their breastfeeding behavior was not affected by treatment [17].

When the study started, fasting serum insulin concentrations in our participants were within normal range for healthy adults, although the values were higher in women with a BMI of >30 kg/m^2^ than those with a BMI of 25–30 kg/m^2^ [25]. Interestingly, the dietary behavior modification significantly reduced these concentrations both at the 12-wk and at the 1-y follow-up. The effect is likely a direct consequence of the 10% loss in body weight, [26] which is an indication that the dietary changes made by the women in response to our intervention, if further sustained, could potentially reduce future risk for type 2 diabetes.

Reductions in inflammation markers have been observed in other studies with weight reductions ≥10% [27]. Although the obese women in LEVA had significantly higher CRP concentrations than overweight women at baseline [25], neither CRP values nor other markers of inflammation were significantly affected by our interventions. This may be explained by the relatively low concentrations of these biomarkers at baseline, the postpartum activation of the immune system [28] and possibly a low precision in the measurements relative to the sample size.

Substantial effects of exercise intervention on maximal oxygen uptake in normal-weight and overweight lactating women have previously been reported [12], [13]. In LEVA, participants reported good compliance with the exercise protocol, but the doubly labeled water method did not show effects of the E treatment on total energy expenditure [17]. As observed by others, a possible explanation is that the women reduced other daily activities [15]. Also, our exercise intervention may not have been sufficiently intense to increase aerobic capacity when performed unsupervised. Finally, as our participants were already quite active (8000 steps/d at baseline), increased energy expenditure may not have been feasible.

This study has several strengths; the most important were the randomized design and the use of state-of-the-art methods for all measurements. The drop-out rate was very low and all 5 women excluded at the 1-y follow-up resulted from new pregnancies. A limitation of this study was the lack of treatment effect from physical activity on total energy expenditure, which limited our ability to draw conclusions regarding effects of increased physical activity level. On the other hand, it is of clinical importance to note that we in line with other studies demonstrate this low feasibility of efforts to increase TEE with exercise treatment. We also observed a trend towards reduced systolic blood pressure from the D treatment that may be of clinical relevance, although our relatively small sample size may have limited our ability to achieve statistical significance. Further, the relatively small sample size may have limited our ability to detect significant interaction effects between the two treatments on several of the outcomes. Finally, our conclusions are restricted to a group of highly educated women with BMI <35 kg/m^2^.

The women who entered this study were overweight or obese before becoming pregnant. This implies that they were already exposed to increased risk for metabolic disease. Their weight status was also important for their risk profile at 12 wk postpartum [25]. In addition, childbearing per se may increase the risk for cardiovascular disease in women [4]–[6]. Here we have shown that this treatment, which led to a 10% sustained weight loss, was also successful in modifying risk markers associated with cardiovascular disease and type 2 diabetes by reducing waist-circumference and fasting insulin as well as increasing HDL-cholesterol concentrations. The postpartum period thus appears to be a window of opportunity for therapeutic dietary interventions among overweight and obese women that may not only reduce weight-related risks in future pregnancies but also their long-term risk for metabolic disease.

## Supporting Information

Checklist S1 **Consort Checklist.**
(DOC)

Protocol S1 **Study Protocol.**
(PDF)
