# Supplementary material for: Diet and Exercise Interventions among Overweight and Obese Lactating Women: Randomized Trial of Effects on Cardiovascular Risk Factors
Source: PLoS One. 2014 Feb 7;9(2):e88250. doi: 10.1371/journal.pone.0088250 (PMC3917884; doi:10.1371/journal.pone.0088250)
Supplement: Protocol S1 — Study Protocol. (PDF) [file pone.0088250.s002.pdf]

GOTHENBURG UNIVERSITY

Short and longterm effects of physical activity and dietary restriction postpartum on women's weight and body composition: a randomized trial

Aim

The aim of the study is to investigate whether, among overweight and obese postpartum women, physical exercise, dietary restrictions or a combination of these treatments leads to a significantly larger reduction in weight over a 1-yr period, compared to those not receiving the intervention. We also will investigate the effects of the intervention on body composition, blood lipids, inflammation markers and cardiovascular fitness. To assure that the treatments do not compromise infant well-being, we will monitor infant growth during the intervention.

Subjects and methods

In total, 68 pregnant women, who were overweight or obese (BMI 25 - 34.9) before pregnancy, will be recruited to join a randomized trial. Women with BMI $\geq$ 35 kg/m^2 (obesity grades II and III) are excluded because their obesity may have causes unrelated to lifestyle and, also, these women may have difficulties following a regular exercise program. All included women should be breastfeeding their infants and plan to do so until at least 6 mo postpartum. Women who are smokers or who deliver twins or have premature labor, low birthweight infant (< 2 500 g) or serious illness on the part of the mother or infant will be excluded. The women are randomized at 10 wk postpartum to four groups according to a 2 x 2 factorial design: (1) only physical exercise, (2) only dietary restrictions, (3) physical exercise + dietary restriction and (4) control. The intervention will last for 12 wk.

The project involves antenatal care clinics in Centrum Väster of Primärvården Göteborg. Suitable women receive extensive information about the study and are invited to participate. Those agreeing are asked to follow the informed consent procedure. At recruitment, women are asked about background information such as age, parity, education, occupation, family history of obesity, own illnesses, self-reported pre-pregnancy weight, weight gain during pregnancy, and the weight and length of their babies at birth.

Baseline measurements are obtained at 10 wk postpartum. At this time, physiological and morphological changes due to pregnancy should have subsided and the breastfeeding should be well established. The baseline measurements include weight, body composition, dietary intake, total energy expenditure, cardiovascular fitness, serum samples (lipids, glucose, insulin, haemoglobin, albumin and the inflammation markers il-6, TNF-a, hs-CRP, SAA and IGF-1). The intervention is initiated once the baseline measurements are complete.

The physical exercise intervention includes 45 min brisk walk with a heart rate monitor 4 days a week at levels representing 60-75% of the maximum heart rate (as estimated in the baseline fitness test). Instructions and follow-up visits are provided by a physical therapist during home visits. To assess overall compliance, physical activity diaries are kept throughout the study and physical activity is measured objectively one week at the beginning, midpoint (6 wk) and end of the intervention. The dietary restriction includes a reduction of total dietary intake of 500 kcal/d compared to baseline. A dietician provides the women in the diet intervention group with individually tailored information about food choices and portion sizes in accordance with the Nordic Nutrition Recommendations (2004) and following behaviour modification therapy. Compliance is measured using weighed food records at the beginning, midpoint (6 wk) and end of the intervention.

Maternal weight is measured 6 wk after the beginning of the intervention to monitor its cumulative effect. At the end of the intervention maternal weight, body composition, total energy expenditure, dietary intake, physical activity, cardiovascular fitness, blood lipids, and inflammation markers are measured to evaluate the short-term effects. One year after the start

of the intervention, the same parameters (except doubly labelled water) are measured again to evaluate the longer-term effects. In between, women are not monitored.

To determine whether the intervention affects the women's ability to breastfeed, infant growth is measured as a proxy for milk production and composition. Infant weight and length will be measured at baseline, at 4, 8 and 12 wk as well as 1 yr after the start of the intervention. At these times, we will also ask the women how they are feeding their babies (ie, exclusive breastfeeding, partial breastfeeding, no breastfeeding) and inquire about infant illnesses. At this last meeting, we will ask women to recall the duration of exclusive breastfeeding. We will also ask if they are still breastfeeding at all and, if not, when they weaned their infant from the breast.

The outcomes changes in weight, BMI, fat mass, fat free mass, cell mass and extra cellular water, cardiovascular fitness, blood lipids and inflammation markers will be studied using repeated measures ANOVA by intervention group in a short term (12 wk post baseline) and longer term (1 y post baseline) perspective. The groups (1) and (3) contribute with information on the effect of physical exercise on the outcomes, compared to the groups (2) and (4). The groups (2) and (3) contribute with information on the effect of dietary restriction on the outcomes, compared to the groups (2) and (4). Finally, the effect of the interaction between physical exercise and dietary restriction will be analyzed in all four groups. For all outcomes statistical adjustments will be made for potentially confounding factors such as age, parity and infant sex. Potential effect modifiers such as pre-pregnant BMI and family history of obesity will be evaluated in multivariate analyses. The expected weight reduction in the intervention group (3) is about 0.5 kg/wk and about 6 kg (± 2 kg; Lovelady et al 2000) during 12 wk. The expected weight reduction in the control group is about 1.5 kg during 12 wk. A sample size of 13 women/group is sufficient to identify a significant difference between these two groups at significance level 0.05 (two-sided) and 90% power, and we will recruit 17 women/group to account for drop-outs. We further propose that the weight reduction in the intervention group remains at 3.0 kg (± 2 kg) at 1 yr post baseline, compared to 1.0 kg for the control group. We have 82% power to detect such a difference.

Ethical considerations

Women are ensured that participation is voluntary and that they can withdraw at any time. Participants' identity information is kept by the principal investigator only and does not appear in any data files. All procedures are safe for postpartum women and are carried out by trained personnel only.

Equipment

Almost all measurements are carried out at the laboratory of Dept of Clinical Nutrition, University of Gothenburg, where equipment representing the latest generation machines for these measurements is available. The only exceptions are the measurements of maternal and infant anthropometry during the intervention, which are assessed during home visits using portable equipment, and the cardiovascular fitness test which is carried out at the Dept of Physiology, University of Gothenburg. Weight of women and children are measured with Seca electronic scales. Infant length is measused using a length carpet.

Cardiovascular fitness is assessed via oxygen uptake while the women are exercising on an exercise bicycle. Body composition is measured with dual energy x-ray absorptiometry (DXA) and multifrequency bioelectrical impedance spectroscopy (BIS), which make it possible to separate not only fat mass and fat-free mass but also cell mass and extracellular

water. Using DXA, long-term energy balance can also be estimated. Basal metabolic rate is measured indirectly using a ventilated hood. Total energy expenditure is measured using doubly labelled water ($^2H_2{}^{18}0$). Urine samples are taken during 2 wk after the ingestion of the oral dose and the samples analyzed using mass spectrometry.

Dietary intake is assessed using 4 days of weighed food records that the women carry out at home according instructions by the dietician. Physical activity is assessed objectively using SenseWear, an armband placed on the upper right arm that measures skin temperature, heat flux from the skin to the environment, galvanic skin response and acceleration along two axes (Mignault et al 2005). Using special algorithms, SenseWear calculates total daily energy expenditure, duration and quantification of physical activity and sleep efficiency and duration.

Women arrive fasting to the Dept of Clinical Nutrition at baseline, at 12 wk and 1 y post-baseline and blood samples are taken by trained staff. Serum levels of cholesterol, LDL, HDL, triglycerides, insulin and inflammation markers are analyzed by the Central Laboratory, Sahlgrenska Hospital, Göteborg.
